# A new caruncle-bearing fanged frog (*Limnonectes*, Dicroglossidae) from Laos and Thailand

**DOI:** 10.3897/zookeys.846.33200

**Published:** 2019-05-16

**Authors:** Somphouthone Phimmachak, Stephen J. Richards, Niane Sivongxay, Sengvilay Seateun, Yodchaiy Chuaynkern, Sunchai Makchai, Hannah E. Som, Bryan L. Stuart

**Affiliations:** 1 National University of Laos, Faculty of Natural Sciences, Department of Biology, P.O. Box 2273, Dong Dok Campus, Vientiane, Laos National University of Laos Vientiane Laos; 2 South Australia Museum, Herpetology Department, Adelaide, South Australia 5000, Australia South Australia Museum Adelaide Australia; 3 Kasetsart University, Faculty of Science, Department of Zoology, Chatuchak, Bangkok, 10900, Thailand Kasetsart University Bangkok Thailand; 4 Khon Kaen University, Faculty of Science, Department of Biology, Khon Kaen, 40002, Thailand Khon Kaen University Khon Kaen Thailand; 5 Natural History Museum, National Science Museum, Thailand, Technopolis, Khlong 5, Khlong Luang, Pathum Thani 12120 Thailand Natural History Museum Pathum Thani Thailand; 6 North Carolina Museum of Natural Sciences, 11 West Jones Street, Raleigh, North Carolina 27601, USA North Carolina Museum of Natural Sciences Raleigh United States of America

**Keywords:** Amphibia, bioacoustics, larval morphology, *
Limnonectes dabanus
*, mitochondrial DNA, Southeast Asia

## Abstract

A new species of the dicroglossid frog genus *Limnonectes* is described from recent and historical museum specimens collected in central and southern Laos and northeastern Thailand. *Limnonectessavan***sp. nov.** has males that bear a caruncle on top of the head, and most closely resembles *L.dabanus* from adjacent southern Vietnam and eastern Cambodia. However, the new species is readily distinguished from *L.dabanus*, and all other caruncle-bearing species of *Limnonectes* in mainland Southeast Asia, by its adult and larval morphology, mitochondrial DNA, and advertisement call. Its description brings the total number of caruncle-bearing species of *Limnonectes* to six.

## Introduction

The dicroglossid frog genus *Limnonectes* Fitzinger, 1843 currently contains 73 species that are distributed from southern China and the Ryukyu Islands of Japan south and eastward to Papua New Guinea (Frost 2019). Most species in the genus exhibit remarkable sexual dimorphism by having males with hypertrophied heads and enlarged odontoid processes on the lower jaw (Lambertz et al. 2014; Rowley et al. 2014; Aowphol et al. 2015), with the latter character earning the genus their colloquial name of “fanged frogs.” Additionally, males of five species in the LimnonectessubgenusElachyglossa Andersson, 1916 bear a swollen or cap-like structure (“caruncle”) on top of their head that consists of a dense pad of connective tissue on the frontoparietal bones (Lambertz et al. 2014): *L.dabanus* (Smith, 1922), *L.gyldenstolpei* (Andersson, 1916), *L.lauhachindai*Aowphol et al., 2015, *L.macrognathus* (Boulenger, 1917) and *L.plicatellus* (Stoliczka, 1873). Caruncle morphology is species-specific (Lambertz et al. 2014; Aowphol et al. 2015), and may be involved in male-male combat (Lambertz et al. 2014; Rowley et al. 2014). Females of most of these species are difficult to distinguish solely from morphology (Aowphol et al. 2015; Phimmachak et al. 2018).

Our collective fieldwork during 1998–2016 at multiple localities in central and southern Laos and northeastern Thailand, and examination of historical museum specimens and the literature (Chan-ard 2003), revealed the presence of a caruncle-bearing *Limnonectes* that could not be assigned to any named species. Males of these “Lao-Thai” specimens generally resemble *L.dabanus*, a species from southern Vietnam and eastern Cambodia (Smith 1922; Stuart et al. 2006; Rowley et al. 2014), but differ in several morphological characters. Herein, we examine adult and larval morphology, mitochondrial DNA, and advertisement calls to test the hypothesis that the “Lao-Thai” specimens represent a distinct species from all other caruncle-bearing *Limnonectes*.

## Materials and methods

### Sampling

Specimens collected in the field were humanely euthanized by immersion in tricaine methanesulfonate (MS-222; Simmons 2015) and fixed in 10% buffered formalin after preserving liver (adults) or the tail (representative larvae) in 20% DMSO-salt saturated storage buffer, RNAlater (Invitrogen), or 95% ethanol. Adult specimens were later transferred to 70% ethanol for permanent storage. Specimens and tissue samples were deposited at the National University of Laos, Faculty of Natural Sciences, Department of Biology (**NUOL**), North Carolina Museum of Natural Sciences (**NCSM**), South Australian Museum (**SAMA**), and Field Museum of Natural History (**FMNH**). Comparative material was examined in the holdings of these institutions and the American Museum of Natural History (**AMNH**), Natural History Museum, London (**NHMUK**), California Academy of Sciences (**CAS**), Muséum national d’Histoire naturelle, Paris (**MNHN**), Museum of Vertebrate Zoology, University of California, Berkeley (**MVZ**), and Zoological Museum Kasetsart University, Bangkok, Thailand (**ZMKU**; Appendix 1). Data for larvae of *L.dabanus* were taken from Rowley et al. [(2014); the larvae of *L.gyldenstolpei*, *L.macrognathus* and *L.lauhachindai* remain unknown (Aowphol et al. 2015)].

### Morphological analyses

Measurements were taken to the nearest 0.1 mm using dial calipers under an ocular microscope. Adult measurements followed Aowphol et al. (2015):

**EYE** eye diameter;

**FTL** pes length from tip of fourth toe to base of inner metatarsal tubercle;

**HDL** head length from tip of snout to rear of jaws;

**HDW** maximum head width;

**HND** manus length from tip of third digit to base of palmar tubercle;

**IML** inner metatarsal tubercle length;

**IMW** inner metatarsal tubercle width;

**IND** internasal distance;

**IOD** interorbital distance, measured as minimum distance between eyes on top of head;

**LAL** forearm length, from elbow to base of palmar tubercle;

**SHK** shank length;

**SNT** snout length from tip of snout to anterior margin of eye;

**SVL** snout-vent length;

**TGH** thigh length, from knee to midline of vent;

**TMP** horizontal diameter of tympanum.

Terminology for the cap-like structure, “caruncle,” followed Lambertz et al. (2014). Specimens were sexed by internal examination of gonads. Staging of eggs and larvae followed Gosner (1960). Larval measurements of body length (BL), tail length (TAL), and total length (TL), and labial tooth row formulae, followed Altig and McDiarmid (1999). Measurements are presented as mean ± standard deviation (SD).

### Phylogenetic analyses

Total genomic DNA was extracted from liver or muscle samples from 53 individuals of *Limnonectes* (Appendix 2) using the ArchivePure DNA Cell/Tissue Kit (5 Prime) or the DNeasy Blood and Tissue Kit (Qiagen). An 551–1,949 nucleotide base pair (bp) fragment of mitochondrial (mt) DNA that encodes parts of the 12S rRNA, tRNA valine, and 16S rRNA genes was amplified by the polymerase chain reaction (PCR; 94–95° C 45s, 53–60° C 30s, 72° C 1 min) for 35 cycles using at least one of four primer combinations: (i) 12L1 (Moriarty and Cannatella 2004) and 16Sbr-3’ (Palumbi 1996), (ii) 16S3L (Chen et al. 2005) and 16Sbr-3’, (iii) 16S3L and H-16SRana (5’-ACAAACGAACCATTAGTAGCG-3’; this study), and/or (iv) 16Sar-5’ (Palumbi 1996) and 16Sbr-3’. Most samples were amplified using the third primer combination. PCR products were sequenced in both directions by direct double strand cycle sequencing using the BigDye Terminator version 3.1 Cycle Sequencing Kit (Applied Biosystems) and the amplifying primers. The internal sequencing primers 16Sar-5’ (Palumbi 1996) and H16SRana-int (Phimmachak et al. 2018) were also used when sequencing the first three primer combinations. Cycle sequencing products were sequenced with a 3130 or 3730 DNA Analyzer (Applied Biosystems). Sequences were edited using Sequencher version 5.4.6 (Gene Codes) and deposited in GenBank under accession numbers MK688558–MK688610. GenBank accession numbers GU934329–31 and GU934337, originally provided by Inger and Stuart (2010), were updated with longer sequences for use in this study.

Newly generated sequences were aligned to the same homologous sequences used by Aowphol et al. (2015) and Phimmachak et al. (2018; Appendix 2). Sequences were aligned using the default parameters in MAFFT v. 7 (Katoh and Standley 2013). The dataset contained representatives of all major clades of *Limnonectes* (based on Evans et al. 2003; Pyron and Wiens 2011), dense sampling of species that are closely related to *L.dabanus*, and the outgroups *Fejervaryalimnocharis* and *Quasipaaspinosa* (based on Pyron and Wiens 2011; Appendix 2). The model of sequence evolution that best described the data (GTR+I+G) was inferred using the Akaike Information Criterion as implemented in jModelTest 2.1.5 (Darriba et al. 2012). Four independent Bayesian analyses were performed using MrBayes 3.2 (Ronquist et al. 2012). In each analysis, four chains were run for 20 million generations using the default priors, the chain temperature was set to 0.1, trees were sampled every 4,000 generations, and the first 25% of trees were discarded as ‘burn-in’. The resulting trace plots were viewed using Tracer v.1.6 (Rambaut et al. 2014). A 50% majority-rule consensus of the post burn-in trees was constructed to calculate the posterior probabilities of nodes. Nodes with posterior probabilities ≥ 0.95 were considered to be statistically supported. Uncorrected pairwise distances were calculated using PAUP* 4.0a (Swofford 2003).

### Bioacoustic analyses

Advertisement calls were recorded from one male (NCSM 76299) at 1935 h on 28 June 2009 that was calling from a wet gully under roots and dead leaves in semi-evergreen forest among a chorus of of approximately 6–10 other males. Calls were recorded under natural conditions at a distance of approximately 0.2 m from the frog using an Edirol R-09 WAVE/MP3 Recorder (96 kHz sampling rate and 24-bit encoding) with a Røde NTG-2 condenser shotgun microphone. Ambient air temperature, relative humidity, and atmospheric pressure were taken immediately after the recording using a Kestrel 3500 hand-held weather meter. Calls were analyzed using Avisoft-SASLab Pro software (Avisoft Bioacoustics). Temporal and spectral parameters of calls, including call duration (ms), inter-call interval (ms), and dominant frequency (kHz), were measured following Köhler et al. (2017).

## Results

### Morphological analyses

Comparisons of “Lao-Thai” specimens to all other *Limnonectes* species having males that bear a caruncle on top of the head revealed consistent differences in body size, shape and position of the head caruncle, shape and length of odontoid processes, dorsal skin texture, toe length, and larval tooth rows.

### Phylogenetic analyses

**Figure 1.**
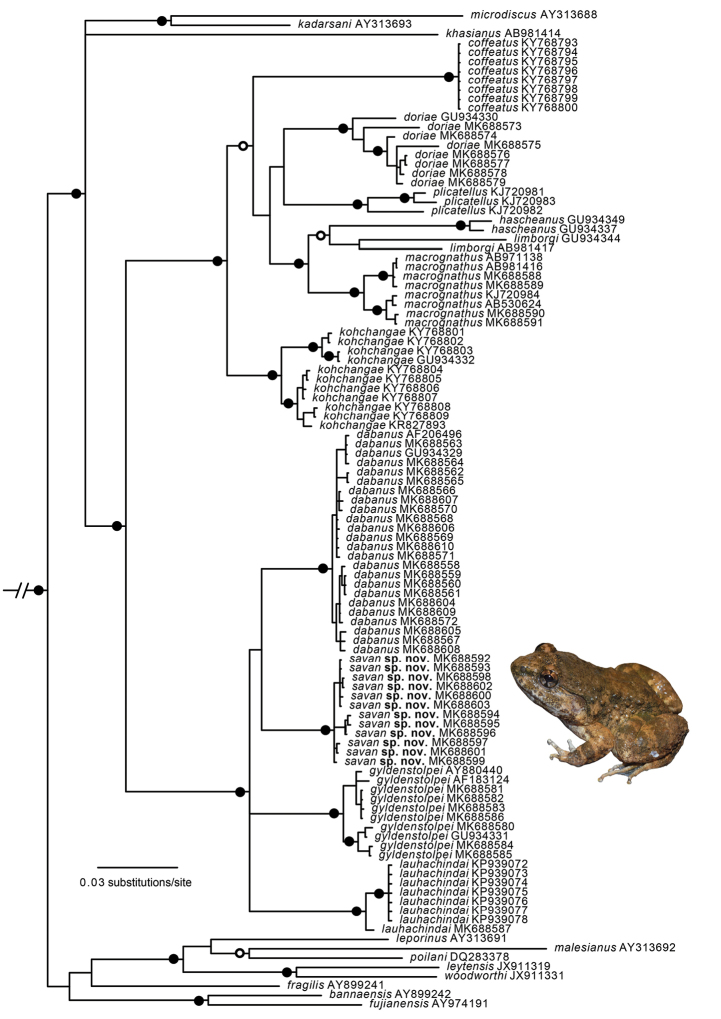
Fifty percent majority-rule consensus phylogram resulting from Bayesian analysis of 2,533 aligned characters of mitochondrial DNA from dicroglossid frogs in the genus *Limnonectes*, and the outgroup taxa *Fejervaryalimnocharis* and *Quasipaaspinosa* (not shown). Black circles at nodes indicate Bayesian posterior probabilities ≥ 0.99, and open circles at nodes indicate Bayesian posterior probabilities ≥ 0.95. Numbers at terminal tips are GenBank accession numbers. Voucher and locality data for sequenced samples are provided in Appendix 2.

The aligned dataset contained 2,533 characters. The standard deviation of split frequencies was 0.005711 among the four Bayesian runs, and the Estimated Sample Sizes (ESS) of parameters were ≥ 1,092. The “Lao-Thai” specimens were recovered as a well-supported monophyletic group (Fig. 1) within a clade containing *L.dabanus*, *L.gyldenstolpei*, and *L.lauhachindai*, but the exact sister taxon relationship of the “Lao-Thai” clade was not resolved (Fig. 1). Thirteen “Lao-Thai” specimens from localities throughout its known range have an uncorrected pairwise divergence of 0–1.05% from each other, but 6.49–7.49% from *L.dabanus* (*n*=24), the species to which it is phenotypically most similar, and to which it was recovered as sister taxon, but without statistical support (Fig. 1).

### Bioacoustic analyses

**Figure 2.**
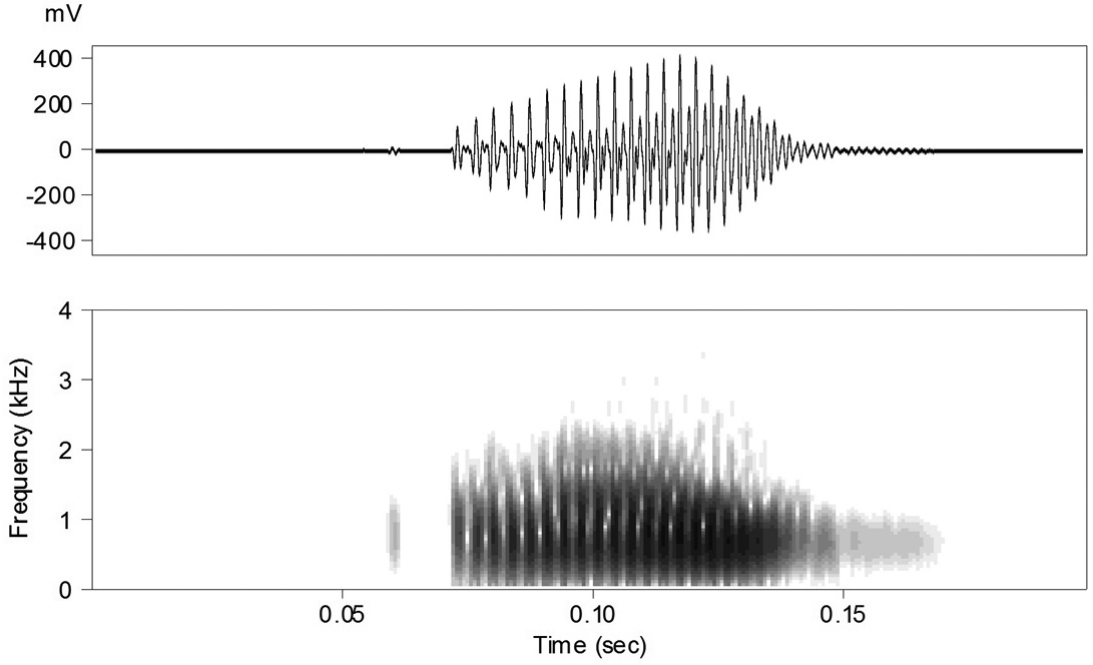
Spectrogram of the advertisement call of an adult male paratype (NCSM 76299) of *Limnonectessavan* sp. nov. from Savannakhet Province, Laos, recorded at an ambient air temperature of 26.3ºC.

Nine advertisement calls from the “Lao-Thai” male specimen NCSM 76299 were recorded at an ambient air temperature of 26.3° C, 100% relative humidity, and atmospheric pressure of 1086.3 hPa. Calls were a single, low-pitched, unmelodic note lasting 57–76 ms (Fig. 2). Notes were finely pulsed, and pulses were not distinguishable to the human ear, but the number of pulses could be determined for four calls. These calls contained 19–21 pulses produced at a rate of 295–312 pulses/s. Calls were amplitude modulated, with amplitude increasing relatively slowly for the first 2/3–3/4 of the call before decreasing rapidly. During several calls, amplitude stabilized or decreased in the middle of the call before increasing again near the end (Fig. 2). The dominant frequency (also the fundamental frequency) was 0.55–0.64 kHz and harmonics were not evident. Calls were repeated relatively slowly and at highly variable intervals, with inter-call intervals (*n*=8) ranging from 5.5–26.8 s. Background calls recorded from other males in the same chorus were of insufficient quality for analysis.

## Species description

On the basis of these corroborated lines of evidence from multiple, independent datasets (larval morphology, adult male morphology, mitochondrial DNA, and male advertisement calls), we hypothesize that the “Lao-Thai” specimens represent a distinct evolutionary lineage that should be recognized as a species, described herein as:

### Limnonectes (Elachyglossa) savan sp. nov.

Taxon classificationAnimaliaAnuraDicroglossidae

http://zoobank.org/673B5D88-212D-40C4-BB8A-3598A1A9E1AD

**Figure 3.**
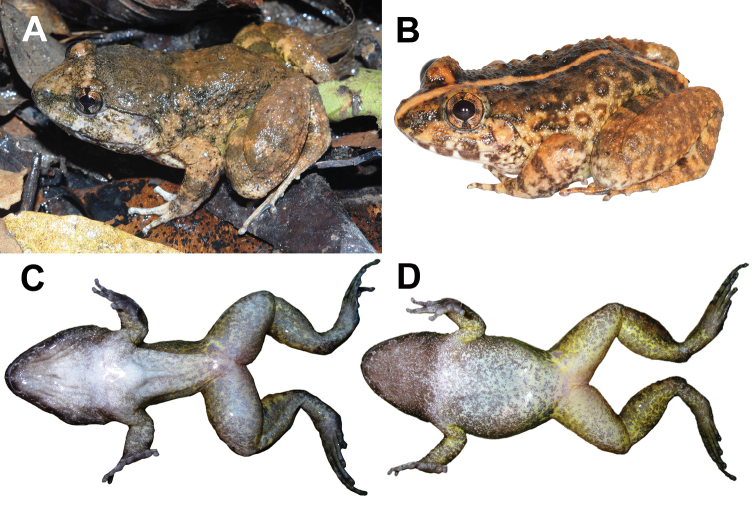
*Limnonectessavan* sp. nov. in life **A** lateral view of holotype male (NCSM 76288) **B** lateral view of paratype female (NUOL 00061) **C** ventral view immediately prior to preservation of paratype male (NCSM 76303) **D** ventral view immediately prior to preservation of paratype female (NCSM 76301).

**Figure 4.**
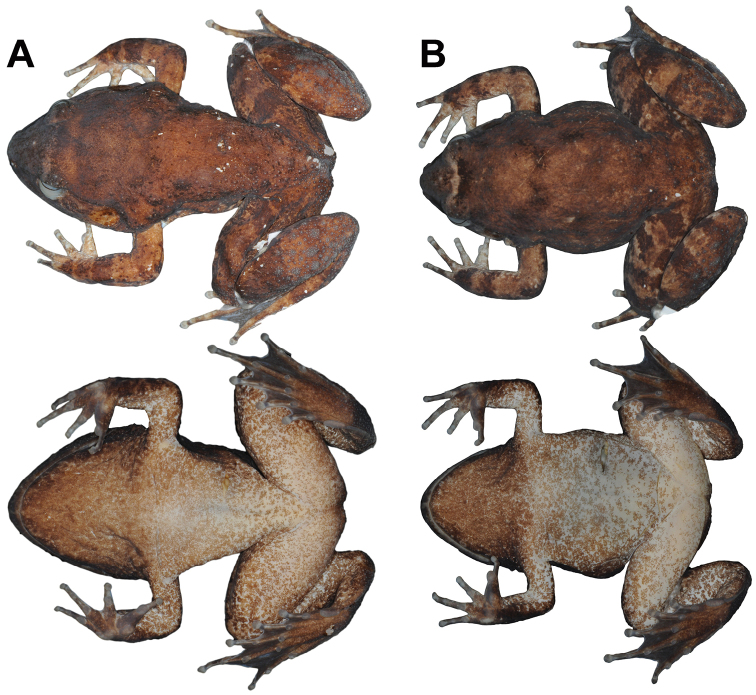
Sexual dimorphism of *Limnonectessavan* sp. nov. in preservative: Dorsal (above) and ventral (below) views of **A** holotype male (NCSM 76288) and **B** paratype female (NCSM 76300).

**Figure 5.**
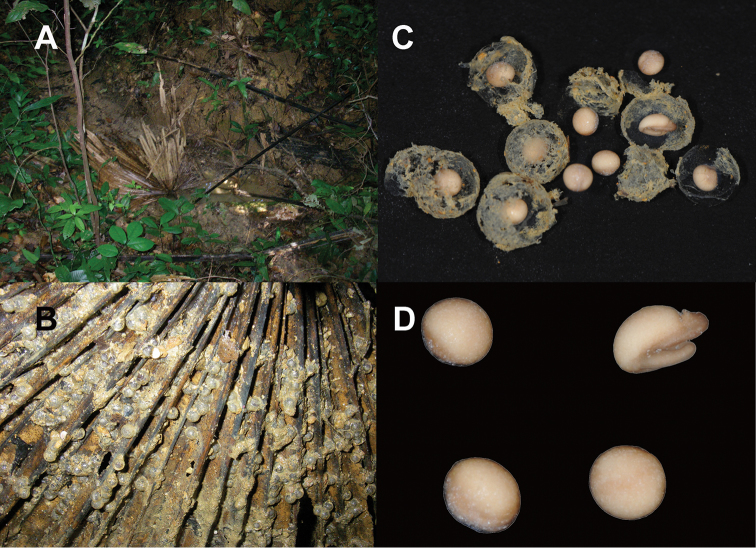
*Limnonectessavan* sp. nov. **A** oviposition site with dead palm frond *in situ* in Savannakhet Province, Laos **B** eggs (NCSM 76494) prior to preservation adhered to underside of dead palm frond that is visible in previous image **C** eggs (NCSM 76494) in preservative with jelly layer **D** eggs (NCSM 76494) in preservative after removal from jelly layer.

**Figure 6.**
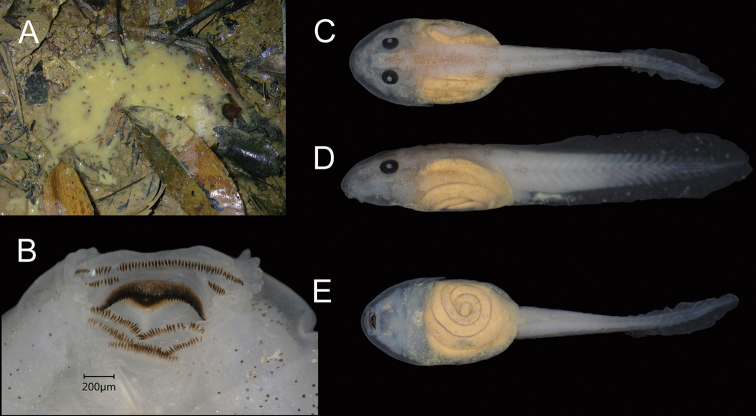
*Limnonectessavan* sp. nov. larvae **A***in situ* in puddle in wet gully in semi-evergreen forest in Savannakhet Province, Laos; one exemplar larva in preservative (NCSM 76492) at Gosner Stage 31, TL 18.4 mm in **B** oral view **C** dorsal view **D** lateral view and **E** ventral view.

Figures 3 , 4 , 5 , 6

Limnonectes sp. Chan-ard, 2003: 120.

#### Holotype.

NCSM 76288 (field tag BLS 12395), adult male (Figs 3, 4), **Laos**, Savannakhet Province, Vilabouli District, Sepon Mining Tenement, Ban Houay Hong Village, Houay Hong Stream, 17.04444°N, 106.12622°E, 254 m elev., under boulder in shallow water of 1–3 m wide swift, rocky stream in semi-evergreen forest, coll.15 November 2008 at 2220 h by Bryan L. Stuart, Somphouthone Phimmachak, Stephen J. Richards, and Niane Sivongxay.

#### Paratypes.

**Laos**, Savannakhet Province, Vilabouli District: NCSM 76287 (one adult male), SAMA R64243 (one juvenile), same data as holotype. NCSM 76294 (one adult female), NCSM 76295, SAMA R64251 (two juveniles), same data as holotype except coll. 04 December 2008. NCSM 76289 (one adult male), SAMA R64244 (one adult female), same data as holotype except Houay Po Stream, 17.04297°N, 106.12503°E, 278 m elev., coll. 18–20 November 2008. SAMA R64245 (one adult male), NCSM 76290, SAMA R64249 (two juveniles), same data as holotype except Ban Nam Pa Village, Houay Hua Tad Stream, 16.96317°N, 106.04661°E, 326 m elev., coll. 22–25 November 2008. SAMA R64246–47 (two adult males), SAMA R64248, NCSM 76291–93 (four juveniles), same data as holotype except Houay Lavi Stream, 16.95653°N, 106.06767°E, 303 m elev., coll. 26 November 2008. SAMA R64250 (one adult female), same data as holotype except Houay Nam Pa Stream, 16.95944°N, 106.04661°E, 280 m elev., coll. 29 November 2008. NCSM 76308 (one adult male), NCSM 76309 (one juvenile), same data as holotype except 17.04120°N, 106.12889°E, 315 m elev., coll. 6 July 2009 by Bryan L. Stuart, Somphouthone Phimmachak, and Niane Sivongxay. NUOL 00092 [formerly NCSM 76298], NCSM 76296, NCSM 76300 (Fig. 4), NCSM 76301 (Fig. 3), NCSM 76304 (five adult females), NUOL 00091 [formerly NCSM 76297], NCSM 76299, NCSM 76302, NCSM 76303 (Fig. 3), NCSM 76305–06 (six adult males), NCSM 76307 (one juvenile), same data as holotype except Nam Sangi River Drainage Basin, 17.02073°N, 106.28625°E, 454 m elev., coll. 25 June–1 July 2009 by Bryan L. Stuart, Somphouthone Phimmachak, and Niane Sivongxay. NCSM 84943 (one adult male), same data as holotype except Ban Namalou Village, 16.93401°N, 105.90562°E, coll. 28 September 2014 by Bryan L. Stuart, Sengvilay Seateun, Niane Sivongxay, Derin Henderson, and Singthong Sanvixay. NUOL 00061 (one adult female; Fig. 3), same data as holotype except Phou Thaengkham Mountain, 16.95279°N, 105.92284°E, coll. 28 September 2014 by Bryan L. Stuart, Sengvilay Seateun, Niane Sivongxay, Derin Henderson, and Singthong Sanvixay.

**Laos**, Khammouan Province, Boualapha District: NCSM 80962 (one juvenile), Xe Bangfay River, 6 km upstream of Ban Pakphanang Village, 17.39972°N, 105.77278°E, coll. 17 March 2002 by Maurice Kottelat. FMNH 255385 (one adult male), FMNH 255386–87 (two juveniles), Hin Nam No National Protected Area, Phou Khaonok Mountain, 17.38333°N, 105.75000°E, 545 m elev., coll. 19–21 February 1998 by Bryan L. Stuart. FMNH 255388 (one adult female), FMNH 255389–90 (two juveniles), same data as FMNH 255385 except 17.33333°N, 105.68333°E, 500 m elev., coll. 23–24 February 1998.

**Laos**, Champasak Province, Pakxong District: NUOL 01151 (one adult female), NUOL 01152–55 (four adult males), Ban Nong Theuam Village, Phou Katam Mountain, Houay Hongkhimin Stream, 15.14461°N, 106.61658°E, 790 m elev., coll. 3 April 2016 by Somphouthone Phimmachak and Sengvilay Seateun. NUOL 01156 (one juvenile), Ban Nam Tuad, downstream of Houay Hongkhimin Stream near road to Attapeu Province, 15.12535°N, 106.63216°E, 503 m elev., coll. 6 April 2016 by Somphouthone Phimmachak and Sengvilay Seateun. NUOL 01157 (one adult male), same data as NUOL 01156 except Xe Katam Waterfall, 15.12355°N, 106.63779°E, 350 m elev.

**Thailand**, Ubon Ratchatani Province, Na Chaluai District: FMNH 266149 (one adult male), Phu Jong-Na Yoi National Park, Huay Luang Noi Stream, 14.43775°N, 105.28006°E, 360 m elev., coll. 15 September 2004 by Bryan L. Stuart, Yodchaiy Chuaynkern, Chatchay Chuechat, and Sunchai Makchai. FMNH 266155 (one adult male), FMNH 266156 (one adult female), same data as FMNH 266149 except hill evergreen forest along road, 14.43850°N, 105.26792°E, 325 m elev., coll. 13 September 2004.

**Thailand**, Ubon Ratchatani Province, Buntharik District: FMNH 266157–58 (two adult females), Phu Jong-Na Yoi National Park, evergreen forest along dirt road, 14.44186°N, 105.30753°E, 400 m elev., coll. 16 September 2004 by Bryan L. Stuart, Yodchaiy Chuaynkern, Chatchay Chuechat, and Sunchai Makchai.

**Thailand**, Ubon Ratchatani Province, Sirindhorn District: FMNH 173514–15 (two juveniles), Forestry Station, Sai Noi River, coll. 23 March 1958 by Edward H. Taylor.

#### Referred specimens.

NCSM 76491 (13 larvae), same data as NUOL 00091. NCSM 76492 (28 larvae), NCSM 76493 (43 larvae), NCSM 76494 (one clutch of 70 eggs), same data as NCSM 76305.

#### Etymology.

The specific epithet *savan* means paradise in the Lao language, and is a commonly used, truncated form of the name for Savannakhet Province, Laos, that contains the holotype and most paratype localities of the new species. The specific epithet *savan* is a noun in apposition.

#### Suggested common names.

Savan Fanged Frog (English), Kop Hone Savan (Lao), Kop Panomdongrak (Thai).

#### Diagnosis.

Assigned to the genus *Limnonectes* on the basis of its inferred phylogenetic position (Fig. 1), the presence of fang-like odontoid processes on the lower jaw (Emerson et al. 2000; Lambertz et al. 2014), and having males with hypertrophied heads (Lambertz et al. 2014). Assigned to the subgenus Elachyglossa (following Ohler and Dubois 1999; Lambertz et al. 2014) on the basis of its close phylogenetic position to the subgenerotype *L.gyldenstolpei* (Fig. 1). A medium-sized *Limnonectes* having the combination of adult males with SVL 39.0–56.2, adult females with SVL 38.9–55.2; males with hypertrophied head; males with interorbital caruncle consisting of low-profile swelling without a free posterior margin, extending from level of anterior margin of eye to level midway between posterior margin of eye and tympanum; odontoid processes on anterior margin of lower jaw larger in males than in females; horizontal diameter of tympanum equal to eye in adult males, ¾ of eye diameter in subadult males, immature males, and females; enlarged, rounded, tubercles on dorsum, becoming more elongated dorsolaterally; dark brown or gray spotting on throat, belly, and ventral surfaces of forelimbs and hindlimbs; and ova with pigmented poles.

#### Description of holotype.

Habitus moderately stocky; body broad anteriorly, tapering to narrow groin. Head broad and depressed, head width equal to head length. Snout obtusely pointed in dorsal view; round, projecting well beyond lower jaw in profile; nostril dorsolateral, much closer to tip of snout than to eye, below canthus; internarial distance 72% of interorbital distance; canthus rostralis indistinct, rounded, slightly constricted behind nostrils; lores concave, oblique; eye diameter 59% of snout length, upper eyelid width 50% of interorbital distance; pineal ocellus visible; tympanum imperfectly circular, not elevated from side of head, annulus visible, tympanum diameter equal to eye diameter and greater than distance between tympanum and eye; small, slit-like vocal sac openings on floor of mouth near lateral margin of tongue; vomerine teeth on two oblique ridges, equidistant to each other as to choanae; two large odontoid processes at front of mandible, triangular, tapered, length subequal to depth of mandible at base of process; median triangular symphysial knob at mandibular symphysis.

Forelimbs robust. Fingers relatively slender, without webbing, with fringe of skin on preaxial and postaxial sides of all fingers, fringes on Fingers II-III movable; tips of fingers rounded, expanded into discs; relative finger lengths II < I < IV < III; distinct, rounded subarticular tubercles, one on Fingers I–II, two on Fingers III–IV; distinct thenar tubercle; two palmar tubercles in contact at base of Fingers II–IV; nuptial pad absent.

Hindlimbs robust. Toes relatively slender; tips of toes rounded, expanded into small discs; relative toe lengths I<II<III=V<IV on right foot, I<II<V<III<IV on left foot; webbing on Toe I to base of disc, on preaxial side of Toe II to level midway between subarticular tubercle and disc and continuing as fringe to base of tip, on postaxial side of Toe II to base of disc, on preaxial side of Toe III to level of distal subarticular tubercle and continuing as fringe to base of disc, on postaxial side of Toe III to base of disc, on preaxial and postaxial sides of Toe IV to level of distal subarticular tubercle and continuing as fringe to base of disc, and on Toe V to base of disc; moveable fringe of skin on outer margins of Toes I and V; distinct fold on distal two-thirds of tarsus; distinct, elongate, oval, inner metatarsal tubercle, length approximately 59% distance between tip of toe I and tubercle; no outer metatarsal tubercle.

Skin on dorsum and flank shagreened with large, irregular, scattered tubercles; tubercles tipped with single, whitish spinule on loreal region, eyelid, lower back near groin, around vent, and ventral surfaces of tibiotarsus and foot; dense clusters of warts (enlarged tubercles), each tipped with numerous whitish spinules, on dorsal surfaces of shank; interorbital caruncle consisting of low-profile swelling without free posterior margin, extending from level of anterior margin of eye to level midway between posterior margin of eye and tympanum, with highest point between eyes; hypertrophied jaw musculature forming two low postorbital swellings on top of head at level of tympanum; distinct supratympanic fold from posterior corner of eye to axilla; rictal gland absent; dorsolateral fold absent; aberrant, triangular skin tag near midline of back; skin on throat with weak longitudinal wrinkles, that on remaining ventral surfaces smooth.

#### Color of holotype in life.

Dorsum tan. Loreal region, tympanic region, and dorsal surfaces of digits whitish gray. Back of head, dorsolateral region, under canthus and dorsoposterior region of tympanum with gray mottling. Dorsal and posterior surfaces of thigh, posterior surface of shank, and groin with yellowish wash. Lips with irregular, broad, gray bars, dorsal surfaces of limbs with cross-bands. Interorbital bar tannish yellow. Iris bronze with black vermiform mottling, black vertical and horizontal bars forming shape of a single plus sign (“+”) over eye (Fig. 3). Ventral surfaces not photographed prior to preservation.

#### Color of paratype male in life.

Based on NCSM 76303. Dorsal surfaces same as holotype except narrow yellow vertebral stripe from tip of snout to vent. Ventral surfaces very light gray with dark gray mottling, only small area near midline of chest nearly immaculate, dark gray mottling becoming denser on ventral surfaces of limbs, nearly uniformly dark gray on ventral surfaces of hands and feet. Inguinal region and ventral surfaces of shank with yellowish wash (Fig. 3).

#### Color of holotype in preservative.

Dorsal surfaces nearly uniformly brown with indistinct, scattered, dark brown mottling, lips with indistinct dark brown bars, and dorsal surfaces of limbs with indistinct dark brown cross-bands. Tympanum and forelimbs with lighter brown than remaining dorsum. Interorbital bar indistinct. Ventral surfaces light brown with dark brown mottling, becoming uniformly dark brown on ventral surfaces of hands and feet (Fig. 4).

#### Description of eggs.

Based on subsample of eggs (NCSM 76494) collected from a larger clutch *in situ* (Fig. 5). Most in Gosner Stage 14, with single jelly layer having diameter of 4.8–5.5 mm (5.1 ± 0.3, *n* = 13), and embryos with a darkly pigmented animal pole having diameter of 2.2–2.5 mm (2.3 ± 0.1, *n* = 13).

#### Description of larvae.

Based on largest individual in series of 28 larvae (NCSM 76492; Fig. 6). Gosner Stage 31, TL 18.4 mm, BL 7.4 mm, TAL 11.0 mm. Body oval in dorsal view, slightly compressed dorsoventrally, maximum body width slightly anterior to level of spiracle. Nares dorsal, without raised rim. Eyes dorsolateral, not visible from below. Spiracular tube single, sinistral on left side, angled slightly dorsally, aperture near midline and projecting posteriorly, approximately midway between snout and end of body. Tail slender, tapering in distal one-fourth to rounded tip, origins of dorsal and ventral fins at end of body, dorsal and ventral fin widest near middle of tail, dorsal fin only slightly deeper than ventral fin. Oral disk ventral, subterminal, width about 39% maximum width of body. Anterior labium with single row of papillae on lateral margins; posterior labium with single row of papillae on lateral and posterior margins; papillae homogenous in length. Labial tooth row formula 2(2)/3(1). A-1 longer than A-2, medial gap in A-2 approximately three-fourth length of A-2. P-1 and P-2 subequal in length, P-3 approximately one-half length of P-1 and P-2. Upper and lower jaw sheaths black with serrated margins, upper sheath without median convexity. In life, dorsum light brown. In preservative, body and tail white with brown mottling on dorsolateral surfaces of body, forming indistinct crossbands on tail. Intestine yellow in dorsal and ventral views. Measurements (TL) of additional larvae (NCSM 76491–93): Gosner Stage 25 11.7–13.1 mm (12.4 ± 0.4, *n* = 15), Gosner Stage 26 12.9–14.5 mm (13.6 ± 0.5, *n* = 13), Gosner Stage 27 14.2–15.8 mm (15.0 ± 0.6, *n* = 13), and Gosner Stage 28 15.5–17.0 mm (16.3 ± 0.8, *n* = 3).

#### Variations.

Females lack caruncle and postorbital swellings (Fig. 3); have narrower heads in dorsal view than males (Table 1; Fig. 4); have relatively smaller tympana than males, with tympanum diameter less than eye diameter (Table 1; Fig. 3); have smaller and shorter odontoid processes than males; have more elongated tubercles on dorsolateral region and flank than males; and contain ova with pigmented poles (Fig. 5).

**Table 1.** Measurements (mm) of types of *Limnonectessavan* sp. nov. and *L.dabanus.* Abbreviations defined in the text. Values are presented as range; mean ± standard deviation (SD).

**Measurement**	*** L. savan ***	*** L. savan ***	*** L. savan ***	*** L. dabanus ***	*** L. dabanus ***
	**Holotype male NCSM 76288**	**Paratype males**	**Paratype females**	**Males**	**Females**
		***n* = 22**	***n* = 14**	***n* = 24**	***n* = 14**
SVL	56.2	39.0–53.6; 45.9 ± 4.8	38.9–55.2; 47.2 ± 5.5	48.8–64.4; 56.9± 4.5	42.3–57.4; 48.2 ± 4.8
HDL	26.8	16.6–26.0; 20.4 ± 2.9	15.7–22.2; 19.2 ± 2.2	21.2–38.0; 26.8 ± 3.5	17.3–24.1; 20.0 ± 1.9
HDW	26.9	17.0–26.1; 20.8 ± 2.7	16.1–22.1; 19.8 ± 2.3	20.8–35.4; 26.5 ± 3.2	17.1–23.9; 19.8 ± 1.8
SNT	9.9	6.1–9.9; 7.8 ± 1.1	6.1–9.2; 7.6 ± 0.9	8.9–14.3; 11.0 ± 1.1	7.0–9.8; 8.2 ± 0.8
EYE	5.8	4.5–6.9; 5.4 ± 0.7	4.4–6.8; 5.5 ± 0.8	5.4–7.3; 6.2 ± 0.5	5.1–7.1; 5.9 ± 0.6
IOD	7.6	3.0–7.5; 5.1 ± 1.2	3.1–4.9; 4.2 ± 0.4	5.2–7.9; 6.4 ± 0.7	3.8–5.3; 4.3 ± 0.3
TMP	5.8	3.4–6.3; 4.7 ± 0.8	3.4–5.5; 4.3 ± 0.7	5.0–9.5; 6.5 ± 1.3	3.7–5.3; 4.4 ± 0.6
IND	5.5	3.6–6.0; 4.6 ± 0.6	3.9–5.6; 4.6 ± 0.5	4.6–7.7; 5.7 ± 0.6	4.1–5.9; 4.7 ± 0.5
SHK	26.9	18.6–25.8; 22.2 ± 2.1	18.9–25.4; 22.5 ± 2.4	23.0–31.1; 27.8 ± 2.7	21.9–29.3; 24.5 ± 2.1
TGH	28.9	18.4–29.1; 23.9 ± 2.8	20.0–28.0; 24.3 ± 3.2	22.0–33.1; 29.1± 3.1	21.9–31.1; 24.8 ± 2.3
LAL	11.0	7.1–10.4; 8.8 ± 0.9	7.7–10.8; 9.2 ± 1.2	8.5–14.1; 11.6 ± 1.3	8.2–11.6; 9.8 ± 0.9
HND	13.8	9.7–14.6; 12.1± 1.3	9.3–14.0; 12.2 ± 1.4	12.4–16.6; 15.1 ± 1.3	11.1–14.1; 12.5 ± 1.0
FTL	26.6	18.8–25.7; 22.3 ± 2.1	18.0–25.4; 22.5± 2.4	22.4–31.4; 27.3 ± 2.7	21.8–27.0; 23.8 ± 1.6
IML	4.1	2.3–4.0; 3.2 ± 0.4	2.9–4.0; 3.4± 0.4	3.0–4.6; 3.8 ± 0.4	2.8–4.2; 3.3 ± 0.4
IMW	1.3	1.0–2.1; 1.3 ± 0.3	0.8–1.6; 1.3 ± 0.2	1.1–2.0; 1.5 ± 0.2	0.9–1.6; 1.1 ± 0.2
TMP:EYE	1.0	0.7–1.1; 0.9 ± 0.1	0.7–0.9; 0.8 ± 0.1	0.8–1.5; 1.0 ± 0.2	0.7–0.8; 0.8 ± 0.1
TMP:SVL	0.1	0.1–0.1; 0.1 ± 0.0	0.1–0.1; 0.1 ± 0.0	0.1–0.2; 0.1 ± 0.0	0.1–0.1; 0.1 ± 0.0

The holotype is the largest male in the type series, with the next largest male (NCSM 76299) having SVL of 53.6 mm. Two paratype males (NCSM 76299, NCSM 76303) have higher-profile caruncles, higher-profile postorbital swellings, and more distinct longitudinal wrinkles on skin of throat than holotype.

Dorsal surfaces are lighter brown, or have more gray mottling, in some specimens than in the holotype. Lip bars on lips and crossbands on dorsal surfaces of limbs more distinct in some specimens than in the holotype. Six paratypes (NCSM 76294, NCSM 76302–04, NUOL 00061, NUOL 00091, NUOL 01153, and SAMA R64247) have a narrow, pale vertebral stripe from tip of snout to vent. Measurements of adults are summarized in Table 1.

#### Distribution, natural history.

**Figure 7.**
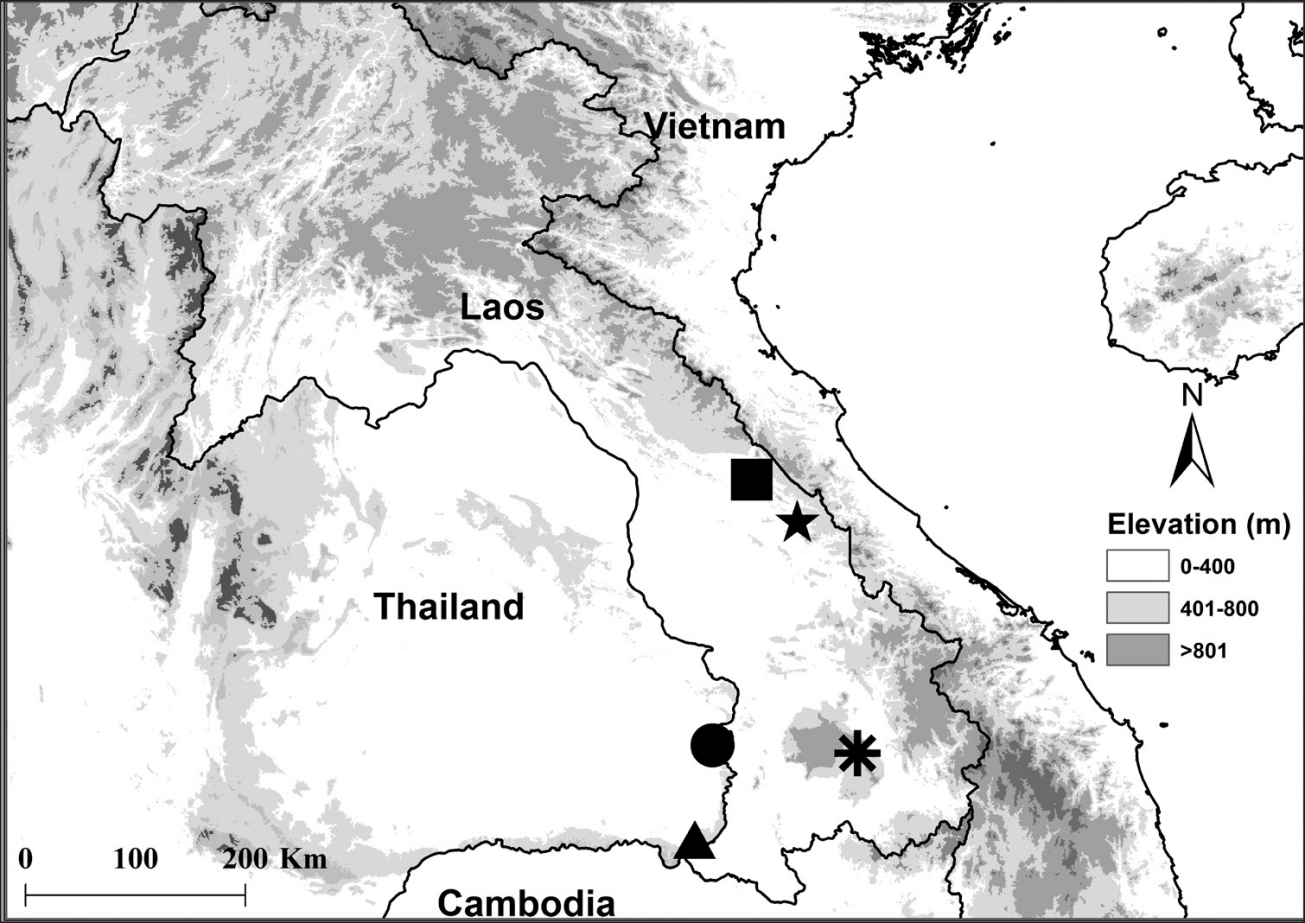
Localities of studied specimens of *Limnonectessavan* sp. nov. at the holotype locality at Savannakhet Province, Vilabouli District, Laos (star) and the paratype localities at Khammouan Province, Boualapha District, Laos (square), Champasak Province, Pakxong District, Laos (asterisk), Ubon Ratchathani Province, Sirindhorn District, Thailand (circle), and Ubon Ratchathani Province, Na Chaluai and Buntharik Districts, Thailand (triangle).

**Figure 8.**
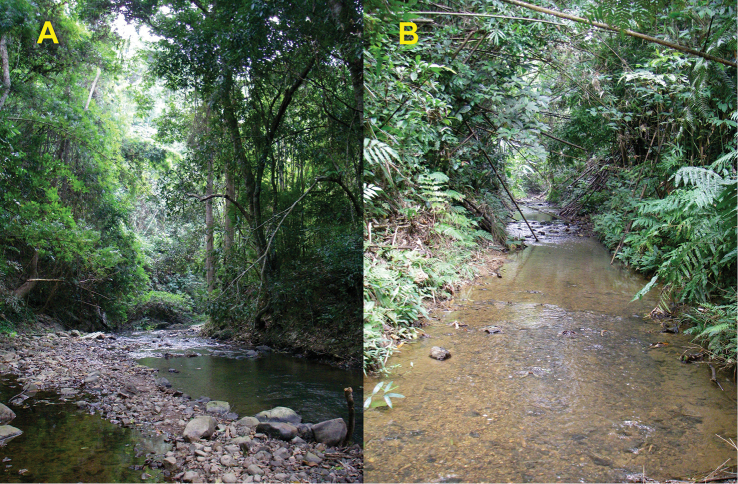
Habitat of *Limnonectessavan* sp. nov. in Savannakhet Province, Vilabouli District, Laos in December 2008 at **A** Houay Khalai Stream, Ban Khalai Village, and **B** Houay Hong Stream, Ban Houay Hong Village.

*Limnonectessavan* is known to occur in central and southern Laos (Khammouan, Savannakhet, and Champasak Provinces), and northeastern Thailand (Ubon Ratchatani; Fig. 7). Chan-ard (2003) also reported it (as *Limnonectes* sp.) from Amnat Charoen Provinces in northeastern Thailand. The species occurs in hill and semi-evergreen forest from 254–790 m elevation, and is usually associated with small (1–3 m wide) streams (Fig. 8); based on 51 specimens sampled at night (1900h–2251h), 38 (74.5%) were found in streams (permanent streams with rocky or sandy substrates, or intermittent streams), nine (17.7%) were found in puddles, two (3.9%) were found in ponds, and two (3.9%) were found on the forest floor, away from an obvious body of water. Nineteen (37.3%) of the 51 specimens were sampled in water, with the remaining 32 individuals (62.7%) found on substrates of soil, leaf litter, rocks or logs.

*Limnonectessavan* breeds in puddles on the forest floor during the rainy season. A chorus of calling males, including paratype male NCSM 76299, was observed in a wet gully under roots and dead leaves in semi-evergreen forest at 1935 h on 28 June 2009. Egg clutch NCSM 76494 was found adhering to the underside of a submerged dead palm frond in a puddle in the same wet gully on 1 July 2009 (Fig. 5). Larvae NCSM 76491 (*n*=13), NCSM 76492 (*n*=28), and NCSM 76493 (*n*=43) were sampled from small puddles (0.2–1 m diameter) in the same wet gully during 28 June–1 July 2009 (Fig. 6).

*Limnonectessavan* occurs in sympatry with *L.lauhachindai* in Ubon Ratchathani Province in northeastern Thailand (Appendix 1), but its geographic distribution appears to be parapatric to that of *L.dabanus* in southern Laos, and to that of *L.gyldenstolpei* in central and southern Laos and northeastern Thailand (Appendix 1).

#### Comparisons.

**Figure 9.**
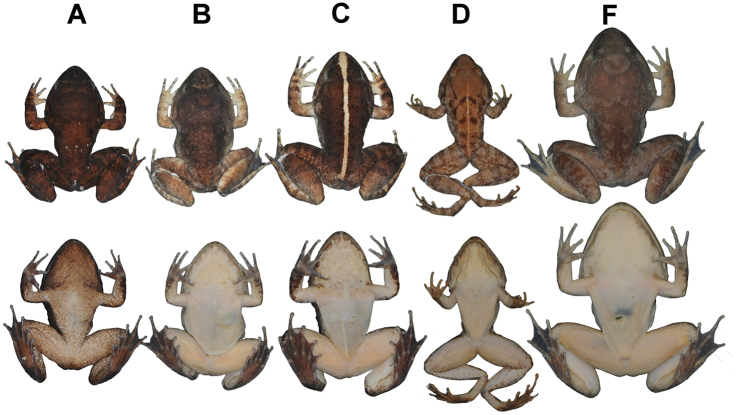
Comparisons of males of four similar, caruncle-bearing species of *Limnonectes* in preservative: Dorsal (above) and ventral (below) views of **A***L.savan* sp. nov. holotype male (NCSM 76288) **B***L.dabanus* (MVZ 258200) with high-profiled caruncle from Ratanakiri Province, Cambodia **C***L.dabanus* (MVZ 258202) with low-profiled caruncle from Ratanakiri Province, Cambodia **D***L.macrognathus* (FMNH 174526) from Nakhon Si Thammarat Province, Thailand **E***L.gyldenstolpei* topotype (ZMKU AM 01143) from Lampang Province, Thailand.

**Figure 10.**
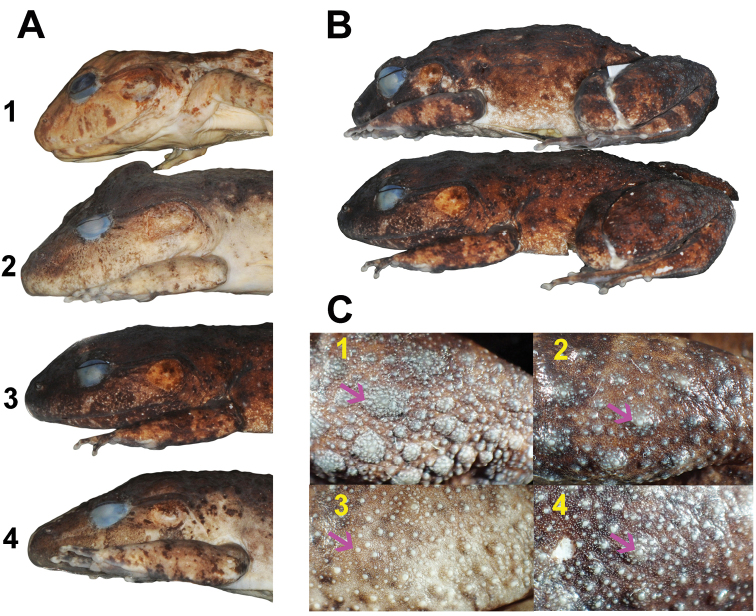
Comparisons of *Limnonectesdabanus* and *L.savan* sp. nov. in preservative **A** lateral view of head of males **A1***L.macrognathus* (FMNH 174526) from Nakhon Si Thammarat Province, Thailand; **A2***L.dabanus* (MVZ 258200) with high-profiled caruncle from Ratanakiri Province, Cambodia **A3***L.savan* sp. nov. holotype (NCSM 76288) **A4***L.dabanus* (MVZ 258202) with low-profiled caruncle from Ratanakiri Province, Cambodia **B** lateral body view of *L.savan* sp. nov. illustrating flank: paratype female (NCSM 76300) above; holotype male (NCSM 76288) below **C** dorsoposterior views of thigh illustrating tubercles (arrows) of **C1***L.savan* sp. nov. holotype male (NCSM 76288) **C2***L.savan* sp. nov. paratype female (NCSM 76300) **C3***L.dabanus* male (MVZ 258200) from Ratanakiri Province, Cambodia **C4***L.dabanus* female (MVZ 258235) from Ratanakiri Province, Cambodia.

*Limnonectessavan* differs from all other species of mainland Southeast Asian *Limnonectes*, except *L.gyldenstolpei*, *L.lauhachindai*, *L.dabanus*, *L.macrognathus*, and *L.plicatellus*, by having mature males with an interorbital caruncle (sensu Lambertz et al. 2014). *Limnonectessavan* differs from these five species by having mature males with interorbital caruncle consisting of low-profile swelling without free posterior margin, with highest point at level between eyes (vs. caruncle U-shaped with free posterior margin in *L.gyldenstolpei* and *L.lauhachindai*, caruncle high-profiled in *L.dabanus*, caruncle high-profiled and horned in *L.plicatellus*, and caruncle with highest point posterior to level of eyes in *L.macrognathus*); and by having dark spotting on ventral surfaces of chest, belly, and limbs in preserved specimens of adults and juveniles of both sexes (vs. these surfaces mostly immaculate in *L.gyldenstolpei*, *L.lauhachindai*, *L.dabanus*, *L.macrognathus*, and *L.plicatellus*; Figs 9, 10).

*Limnonectessavan* further differs from *L.gyldenstolpei*, *L.lauhachindai*, *L.dabanus*, and *L.macrognathus* by having relative toe lengths I<II<III=V<IV, with the tips of Toes III and V reaching the base of the distal subarticular tubercle on Toe IV (vs. relative toe lengths I<II<V<III<IV, with the tip of Toe III shorter not reaching the distal subarticular tubercle on Toe IV in *L.gyldenstolpei*, *L.lauhachindai*, *L.dabanus*, and *L.macrognathus*). *Limnonectessavan* further differs from *L.plicatellus* by having males with much larger body size (SVL ≤ 43.0 in *L.plicatellus*; Boulenger 1920; Taylor 1962; Chan-ard 2003; Lambertz et al. 2014) and by lacking dorsal rugosities arranged in distinct, longitudinal rows parallel to the body axis (vs. present in *L.plicatellus*).

**Figure 11.**
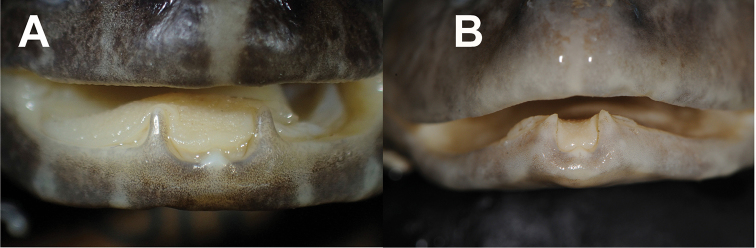
Comparisons of adult male odontoid processes in **A***L.savan* sp. nov. (NUOL 01153) from Champasak Province, Laos, and **B***L.dabanus* (NCSM 80375) from Binh Thuan Province, Vietnam.

*Limnonectessavan* is phenotypically most similar and phylogenetically most closely related (Fig. 1), to *L.dabanus*. The new species further differs from *L.dabanus* by having mature males with two large, tapered odontoid processes of length subequal to depth of mandible at base of process (vs. odontoid processes much less tapered and with length less than one-half depth of mandible at base of process in *L.dabanus*; Fig. 11); by having mature males with TMP = EYE (vs. TMP > EYE in *L.dabanus*); by having the dorsal surfaces of shank with dense clusters of warts, each tipped with numerous whitish spinules (vs. warts and tubercles less distinct and lower in profile, with more homogeneous distributions of whitish spinules in *L.dabanus*); by having larvae with A-1 longer than A-2 (vs. A-1 and A-2 subequal in length in *L.dabanus*), with medial gap in A-2 approximately three-fourths length of A-2 (vs. approximately one-half length of A-2 in *L.dabanus*), and having P-1 and P-2 subequal in length (vs. P-1 longer than P-2 in *L.dabanus*); and by having much shorter calls of 57–74 ms (vs. 141–197 ms in *L.dabanus*; Rowley et al. 2014).

Male secondary sexual characters are unknown in *L.khammonensis* (Smith 1929), which is known only from the female holotype, but females of *L.savan* differ by having a distinct tympanum (indistinct in *L.khammonensis*); having larger body size, with SVL 38.9–55.2 (vs. gravid holotype female SVL 37.5 in *L.khammonensis*); and less toe webbing (vs. Toe IV webbed to distal subarticular tubercle, continuing as fringe to base of disc, and all remaining toes webbed to base of disc in *L.khammonensis*).

## Discussion

New species of *Limnonectes* continue to be discovered and described in mainland Southeast Asia (e.g. Matsui et al. 2010; McLeod et al. 2012; Aowphol et al. 2015; Pham et al. 2018; Phimmachak et al. 2018), and the description of *L.savan* brings the number of named, caruncle-bearing *Limnonectes* species to six. Additional fieldwork is needed to clarify the geographic distribution of the new species, in particular, any co-occurrence with the morphologically-similar (and, potentially, sister taxon) *L.dabanus* in southern Laos.

This study found that *L.savan* is phylogenetically related to the caruncle-bearing species *L.dabanus*, *L.gyldenstolpei*, and *L.lauhachindai* (Fig. 1), but the exact relationships among those species remained unresolved owing to a lack of statistical support (Fig. 1). Ohler and Dubois (1999) recognized ElachyglossaAndersson, 1916 at the subgenus rank for eight species of *Limnonectes*, including *L.dabanus*, *L.gyldenstolpei* (the subgenerotype species), and *L.lauhachindai*, the three closest relatives to *L.savan* (Fig. 1). Lambertz et al. (2014) restricted the subgenus Elachyglossa to the four caruncle-bearing species of *Limnonectes* that were recognized at the time (*L.dabanus*, *L.gyldenstolpei*, *L.macrognathus*, and *L.plicatellus*), but they did not study the caruncle-lacking species *L.hascheanus* (Stoliczka, 1870), *L.limborgi* (Sclater, 1892), and *L.doriae* (Boulenger, 1887) that render the caruncle-bearing species to be non-monophyletic (Fig. 1; Aowphol et al. 2015; Phimmachak et al. 2018). Hence, we are confident in placing *L.savan* in the subgenus Elachyglossa, but recognize that determining the full taxonomic content of the subgenus requires additional study. The generation of a larger (ideally, multi-locus) molecular dataset would likely resolve the sister relationship of *L.savan* and the conflicting hypotheses of relationships among the caruncle-bearing *Limnonectes* species that have been generated in recent studies (Lambertz et al. 2014; Aowphol et al. 2015; Phimmachak et al. 2018), including the taxonomic content of the subgenus Elachyglossa.

Finally, the taxonomic identity of the Lao-endemic *L.khammonensis*, known only from the female holotype specimen taken near Ban Na Pe (“Napé; Smith 1929) in Bolikhamxay Province, ca. 125 air-km north of the northernmost known locality of *L.savan* in Khammouan Province, Laos, needs to be resolved. It is clear on the basis of adult female morphology that *L.khammonensis* is not conspecific with *L.savan*. However, the lack of known males precludes knowing if *L.khammonensis* also bears a caruncle, and might be phylogenetically closely related to *L.savan*. Fieldwork at the type locality of *L.khammonensis* to obtain additional material of this taxon is warranted to facilitate future biodiversity research on *Limnonectes* in the region.

## Supplementary Material

XML Treatment for Limnonectes (Elachyglossa) savan

## Supplementary materials

Supplementary material 1 Appendix 1. Comparative specimens examined Data type: species data This dataset is made available under the Open Database License (http://opendatacommons.org/licenses/odbl/1.0/). The Open Database License (ODbL) is a license agreement intended to allow users to freely share, modify, and use this Dataset while maintaining this same freedom for others, provided that the original source and author(s) are credited.Robert Mesibov

Supplementary material 2 Appendix 2. *Fejervarya*, *Quasipaa*, and *Limnonectes* 16S sequences used in the phylogenetic analysis Data type: species data This dataset is made available under the Open Database License (http://opendatacommons.org/licenses/odbl/1.0/). The Open Database License (ODbL) is a license agreement intended to allow users to freely share, modify, and use this Dataset while maintaining this same freedom for others, provided that the original source and author(s) are credited.Robert Mesibov
