# Supplementary material for: A new caruncle-bearing fanged frog (Limnonectes, Dicroglossidae) from Laos and Thailand
Source: Zookeys. 2019 May 16;846:133–56. doi: 10.3897/zookeys.846.33200 (PMC6533239; doi:10.3897/zookeys.846.33200)
Supplement: Supplementary material 1 [file zookeys-846-133-s001.docx]

**Appendix 1**

**Comparative specimens examined**

An asterisk (*) indicates that the specimen was not included in Table 1 owing to an incomplete set of measurements.

***Limnonectes dabanus***

CAMBODIA: Mondolkiri Province: FMNH 261924, FMNH 261929, FMNH 261932 (three adult males); Ratanakiri Province: MVZ 258200, MVZ 258202, MVZ 258228, MVZ 258230, MVZ 258234, MVZ 258236, MVZ 258238, MVZ 258240–43, MVZ 58247–50 (15 adult males), MVZ 258201, MVZ 258231, MVZ 258233, MVZ 258235, MVZ 258237, MVZ 258239, MVZ 258244, MVZ 258246 (eight adult females); Stung Treng Province, FMNH 262919 (one adult female); Tbong Khmum Province: MNHN 1948.0126* (adult male holotype of *Rana toumanoffi*). LAOS: Champasak Province: FMNH 258145, FMNH 258147–48 (three adult males), FMNH 258146, FMNH 258149–50, FMNH 258178–79 (five adult females). VIETNAM: Binh Thuan Province: AMNH A191966–67, NCSM 80375 (three adult males); Ninh Thuan Province (“Daban”): BMNH 1947.2.1.19* (adult male syntype), BMNH 1974.1807* (adult female syntype).

***Limnonectes khammonensis***

LAOS: Bolikhamxay Province: BMNH 1928.6.29.14 (adult female holotype).

***Limnonectes macrognathus***

MYANMAR: “Karin Hills” (province unknown): BMNH 1947.2.1.61 (adult male syntype). THAILAND: Nakhon Si Thammarat Province: FMNH 174526, FMNH 270104 (two adult males), FMNH 270105 (one adult female).

***Limnonectes gyldenstolpei***

CAMBODIA: Kampong Speu Province: NCSM 79548–51, NCSM 79554 (five adult males), NCSM 79552–53, NCSM 79555 (three adult females). LAOS: Luang Phabang Province: NCSM 79308 (one adult male), NCSM 79298, NUOL 00075 (two adult females); Vientiane Province: NCSM 79063 (one adult male); Xaignabouli Province: NCSM 79060–61, NCSM 79301, NCSM 79304, NCSM 79306–07, NCSM 79310, NCSM 79313, NUOL 00071–72 (10 adult males), NCSM 79058, NCSM 79302, NCSM 79305, NCSM 79309, NCSM 79312 (five adult females). THAILAND: Lampang Province: ZMKU AM 01143 (one adult male topotype), ZMKU AM 01144 (one adult female topotype).

***Limnonectes lauhachindai***

THAILAND: Ubon Ratchathani Province: NCSM 80222 (adult male holotype), NCSM 81269, ZMKU AM 00552–53, ZMKU AM 00586–92, ZMKU AM 01104-09, ZMKU AM 01111, FMNH 266148/THNHM 05185, FMNH 266154/THNHM 05189 (19 adult male paratypes), FMNH 266147/THNHM 05184, FMNH 266150/THNHM 05025, FMNH 266151/THNHM 05026, FMNH 266152/THNHM 05187 (four adult female paratypes).

***Limnonectes plicatellus***

MALAYSIA: Selangor State: FMNH 186563–80 (18 adult males)
