# Supplementary material for: A new caruncle-bearing fanged frog (Limnonectes, Dicroglossidae) from Laos and Thailand
Source: Zookeys. 2019 May 16;846:133–56. doi: 10.3897/zookeys.846.33200 (PMC6533239; doi:10.3897/zookeys.846.33200)
Supplement: Supplementary material 2 [file zookeys-846-133-s002.docx]

**Appendix 2**

*Fejervarya*, *Quasipaa*, and *Limnonectes* 16S sequences used in the phylogenetic analysis. Institutional abbreviations of vouchers follow Sabaj-Pérez (2016), with the additions of PWRC = Phu Luang Wildlife Research Centre Museum and ZNAC = Anhui Normal University.

| **Species** | **GenBank No.** | **Voucher** | **Locality** | **Source** |  |
| --- | --- | --- | --- | --- | --- |
| *F. limnocharis* | NC_005055 | None | China, Yancheng | Liu et al. (2005) |  |
| *Q. spinosa* | NC_013270 | None | China, Zhejiang Prov., Jinhua | Zhou et al. (2009) |  |
| *L. bannaensis* | AY899242 | None | China, Yunnan Prov., Simao | Zhang et al. (2009) |  |
| *L. coffeatus* | KY768794 | FMNH 258440 | Laos, Champasak Prov., Pakxong Dist. | Phimmachak et al. (2018) |  |
| *L. coffeatus* | KY768795 | FMNH 258441 | Laos, Champasak Prov., Pakxong Dist. | Phimmachak et al. (2018) |  |
| *L. coffeatus* | KY768796 | NCSM 77785 | Laos, Champasak Prov., Pakxong Dist. | Phimmachak et al. (2018) |  |
| *L. coffeatus* | KY768797 | NCSM 77786 | Laos, Champasak Prov., Pakxong Dist. | Phimmachak et al. (2018) |  |
| *L. coffeatus* | KY768798 | NCSM 77787 | Laos, Champasak Prov., Pakxong Dist. | Phimmachak et al. (2018) |  |
| *L. coffeatus* | KY768799 | NCSM 77788 | Laos, Champasak Prov., Pakxong Dist. | Phimmachak et al. (2018) |  |
| *L. coffeatus* | KY768793 | NCSM 77944 | Laos, Champasak Prov., Pakxong Dist. | Phimmachak et al. (2018) |  |
| *L. coffeatus* | KY768800 | NUOL 00060 | Laos, Champasak Prov., Pakxong Dist. | Phimmachak et al. (2018) |  |
| *L. dabanus* | MK688558 | FMNH 258146 | Laos, Champasak Prov., Pakxong Dist. | This study |  |
| *L. dabanus* | MK688559 | FMNH 258147 | Laos, Champasak Prov., Pakxong Dist. | This study |  |
| *L. dabanus* | MK688560 | FMNH 258148 | Laos, Champasak Prov., Pakxong Dist. | This study |  |
| *L. dabanus* | MK688561 | FMNH 258149 | Laos, Champasak Prov., Pakxong Dist. | This study |  |
| *L. dabanus* | MK688562 | FMNH 261924 | Cambodia, Mondolkiri Prov., Pichrada Dist. | This study |  |
| *L. dabanus* | MK688563 | FMNH 261925 | Cambodia, Mondolkiri Prov., Pichrada Dist. | This study |  |
|  |  |  |  |  |  |
| *L. dabanus* | GU934329 | FMNH 261937 | Cambodia, Mondolkiri Prov., Pichrada Dist. | Inger and Stuart (2010) |  |
| *L. dabanus* | MK688564 | FMNH 261939 | Cambodia, Mondolkiri Prov., Pichrada Dist. | This study |  |
| *L. dabanus* | MK688604 | FMNH 262744 | Cambodia, Stung Treng Prov., Siem Pang Dist. | This study |  |
| *L. dabanus* | MK688605 | FMNH 262745 | Cambodia, Stung Treng Prov., Siem Pang Dist. | This study |  |
| *L. dabanus* | MK688565 | FMNH 262747 | Cambodia, Mondolkiri Prov., O’Rang Dist. | This study |  |
| *L. dabanus* | MK688566 | FMNH 262908 | Cambodia, Ratanakiri Prov., Ta Veng Dist. | This study |  |
| *L. dabanus* | MK688567 | FMNH 262921 | Cambodia, Stung Treng Prov., Siem Pang Dist. | This study |  |
| *L. dabanus* | MK688568 | MVZ 258200 | Cambodia, Ratanakiri Prov., Veunsai Dist. | This study |  |
| *L. dabanus* | MK688606 | MVZ 258202 | Cambodia, Ratanakiri Prov., Veunsai Dist. | This study |  |
| *L. dabanus* | MK688569 | MVZ 258228 | Cambodia, Ratanakiri Prov., Veunsai Dist. | This study |  |
| *L. dabanus* | MK688607 | MVZ 258236 | Cambodia, Ratanakiri Prov., Veunsai Dist. | This study |  |
| *L. dabanus* | MK688570 | MVZ 258237 | Cambodia, Ratanakiri Prov., Veunsai Dist. | This study |  |
| *L. dabanus* | MK688608 | MVZ 258240 | Cambodia, Ratanakiri Prov., Veunsai Dist. | This study |  |
| *L. dabanus* | MK688609 | MVZ 258244 | Cambodia, Ratanakiri Prov., Veunsai Dist. | This study |  |
| *L. dabanus* | MK688610 | MVZ 258247 | Cambodia, Ratanakiri Prov., Veunsai Dist. | This study |  |
| *L. dabanus* | MK688571 | MVZ 258249 | Cambodia, Ratanakiri Prov., Veunsai Dist. | This study |  |
| *L. dabanus* | MK688572 | MVZ 258250 | Cambodia, Ratanakiri Prov., Veunsai Dist. | This study |  |
| *L. dabanus* | AF206496 | ROM 22081 | Vietnam, Yok Don | Chen et al. (2005) |  |
| *L. doriae* | GU934330 | CAS 208425 | Myanmar, Pegu State, Bago Yoma | Inger and Stuart (2010) |  |
| *L. doriae* | MK688573 | CAS 211632 | Myanmar, Rakhine State, Rakhine Yoma | This study |  |
| *L. doriae* | MK688574 | CAS 229581 | Myanmar, Tanintharyi Div., Kawthaung Dist. | This study |  |
| *L. doriae* | MK688575 | CAS 229714 | Myanmar, Tanintharyi Div., Dewei Dist. | This study |  |
| *L. doriae* | MK688576 | FMNH 268506 | Thailand, Surat Thani Province, Kaeng Krung | This study |  |
| *L. doriae* | MK688577 | FMNH 268509 | Thailand, Surat Thani Province, Kaeng Krung | This study |  |
| *L. doriae* | MK688578 | FMNH 270111 | Thailand, Surat Thani Province, Khao Sok | This study |  |
| *L. doriae* | MK688579 | FMNH 270113 | Thailand, Surat Thani Province, Khao Sok | This study |  |
| *L. fragilis* | AY899241 | ZNAC 11006 | Not available | GenBank submission |  |
| *L. fujianensis* | AY974191 | Not available | Not available | GenBank submission |  |
| *L. gyldenstolpei* | MK688580 | FMNH 257333 | Cambodia, Siem Reap Prov., Bante Sre Dist. | This study |  |
| *L. gyldenstolpei* | GU934331 | FMNH 266203 | Thailand, Sa Kaeo Prov., Mueang Sa Kaeo Dist. | Inger and Stuart (2010) |  |
| *L. gyldenstolpei* | MK688581 | NCSM 79297 | Laos, Luang Phabang Prov., Luang Phabang Dist. | This study |  |
| *L. gyldenstolpei* | MK688582 | NCSM 79310 | Laos, Xaignabouli Prov., Xaignabouli Dist. | This study |  |
| *L. gyldenstolpei* | MK688583 | NCSM 79312 | Laos, Xaignabouli Prov., Paklay Dist. | This study |  |
| *L. gyldenstolpei* | MK688584 | NCSM 79549 | Cambodia, Kampong Speu Prov., Aural Dist. | This study |  |
| *L. gyldenstolpei* | MK688585 | NCSM 79555 | Cambodia, Kampong Speu Prov., Aural Dist. | This study |  |
| *L. gyldenstolpei* | MK688586 | NUOL 00075 | Laos, Luang Phabang Prov., Nan Dist. | This study |  |
| *L. gyldenstolpei* | AY880440 | MNHN 1998.4150 | Vietnam | Delorme et al. (2004) |  |
| *L. gyldenstolpei* | AF183124 | PWRC 002 | Thailand, Loei Prov., Phu Luang | Emerson et al. (2000) |  |
| *L. hascheanus* | GU934337 | FMNH 270118 | Thailand, Nakhon Si Thammarat Prov., Khao Luang | Inger and Stuart (2010) |  |
| *L. hascheanus* | GU934349 | LSUHC 6777 | Malaysia, Penang | Inger and Stuart (2010) |  |
| *L. kadarsani* | AY313693 | LSUMZ 81722 | Indonesia, Lombok Island | Evans et al. (2003) |  |
| *L. kohchangae* | KY768801 | FMNH 263198 | Cambodia, Kampot Prov., Kampot Dist. | Phimmachak et al. (2018) |  |
| *L. kohchangae* | KY768802 | FMNH 263201 | Cambodia, Kampot Prov., Kampot Dist. | Phimmachak et al. (2018) |  |
| *L. kohchangae* | GU934332 | FMNH 263210 | Cambodia, Kampong Speu Prov., Phnom Sruoch Dist. | Inger and Stuart (2010) |  |
| *L. kohchangae* | KY768803 | FMNH 263212 | Cambodia, Kampong Speu Prov., Phnom Sruoch Dist. | Phimmachak et al. (2018) |  |
| *L. kohchangae* | KY768808 | NCSM 79542 | Cambodia, Kampong Speu Prov., Aural Dist. | Phimmachak et al. (2018) |  |
| *L. kohchangae* | KY768809 | NCSM 79546 | Cambodia, Kampong Speu Prov., Aural Dist. | Phimmachak et al. (2018) |  |
|  |  |  |  |  |  |
| *L. kohchangae* | KR827893 | MNHN 2003.0317 | Cambodia, Koh Kong, Cham Not Tapan | Grosjean et al. (2015) |  |
| *L. kohchangae* | KY768804 | ZMKU AM 01155 | Thailand, Trat Prov., Ko Chang Dist. | Phimmachak et al. (2018) |  |
| *L. kohchangae* | KY768806 | ZMKU AM 01156 | Thailand, Trat Prov., Ko Chang Dist. | Phimmachak et al. (2018) |  |
| *L. kohchangae* | KY768805 | ZMKU AM 01157 | Thailand, Trat Prov., Ko Chang Dist. | Phimmachak et al. (2018) |  |
| *L. kohchangae* | KY768807 | ZMKU AM 01158 | Thailand, Trat Prov., Ko Chang Dist. | Phimmachak et al. (2018) |  |
| *L. khasianus* | AB981414 | KUHE 23158 | Thailand, Narathiwat Prov., Bala | Matsui et al. (2014) |  |
| *L. lauhachindai* | MK688587 | FMNH 266153 | Thailand, Ubon Ratchathani Prov., Na Chaluai Dist. | This study |  |
| *L. lauhachindai* | KP939072 | NCSM 80222 | Thailand, Ubon Ratchathani Prov., Sirindhorn Dist. | Aowphol et al. (2015) |  |
| *L. lauhachindai* | KP939078 | NCSM 81269 | Thailand, Ubon Ratchathani Prov., Sirindhorn Dist. | Aowphol et al. (2015) |  |
| *L. lauhachindai* | KP939073 | ZMKU AM 01104 | Thailand, Ubon Ratchathani Prov., Sirindhorn Dist. | Aowphol et al. (2015) |  |
| *L. lauhachindai* | KP939074 | ZMKU AM 01105 | Thailand, Ubon Ratchathani Prov., Sirindhorn Dist. | Aowphol et al. (2015) |  |
| *L. lauhachindai* | KP939075 | ZMKU AM 01106 | Thailand, Ubon Ratchathani Prov., Sirindhorn Dist. | Aowphol et al. (2015) |  |
| *L. lauhachindai* | KP939076 | ZMKU AM 01107 | Thailand, Ubon Ratchathani Prov., Sirindhorn Dist. | Aowphol et al. (2015) |  |
| *L. lauhachindai* | KP939077 | ZMKU AM 01109 | Thailand, Ubon Ratchathani Prov., Sirindhorn Dist. | Aowphol et al. (2015) |  |
| *L. leporinus* | AY313691 | AMNH 167165 | Indonesia, Kalimantan Timor Prov., Kutai | Evans et al. (2003) |  |
| *L. leytensis* | JX911319 | USNM 222545 | Philippines, Leyte Island | Oaks et al. (2013) |  |
| *L. limborgi* | GU934344 | FMNH 262817 | Cambodia, Mondolkiri Prov., Samling | Inger and Stuart (2010) |  |
| *L. limborgi* | AB981417 | KUHE 15614 | Malaysia, Janda Baik | Matsui et al. (2014) |  |
| *L. macrognathus* | MK688588 | FMNH 268503 | Thailand, Surat Thani Province, Kaeng Krung | This study |  |
| *L. macrognathus* | MK688589 | FMNH 268505 | Thailand, Surat Thani Province, Kaeng Krung | This study |  |
| *L. macrognathus* | KJ720984 | FMNH 270104 | Thailand, Nakhon Si Thamarat Prov., Khao Luang | Lambertz et al. (2014) |  |
| *L. macrognathus* | MK688590 | FMNH 270114 | Thailand, Surat Thani Province, Khao Sok | This study |  |
| *L. macrognathus* | MK688591 | FMNH 270115 | Thailand, Surat Thani Province, Khao Sok | This study |  |
| *L. macrognathus* | AB530624 | IABHU 21120 | Malaysia, Langkawi Island | Hasan et al. (2014) |  |
| *L. macrognathus* | AB971138 | KUHE 23923 | Thailand, Ranong Prov. | Matsui and Nishikawa (2014) |  |
| *L. macrognathus* | AB981416 | KUHE 23923 | Thailand, Ranong Prov. | Matsui et al. (2014) |  |
| *L. malesianus* | AY313692 | Not available | Malaysia, Sarawak Prov., Gunung Buda | Evans et al. (2003) |  |
| *L. microdiscus* | AY313688 | LSUMZ 81739 | Indonesia, Java, Sukabumi | Evans et al. (2003) |  |
| *L. plicatellus* | KJ720981 | LSUHC 6710 | Malaysia, Pulau Pinang, Empangan Air Hitam | Lambertz et al. (2014) |  |
| *L. plicatellus* | KJ720982 | LSUHC 6582 | Malaysia, Selangor Dist., Gombak Swamp | Lambertz et al. (2014) |  |
| *L. plicatellus* | KJ720983 | LSUHC 4001 | Malaysia, Selangor Dist., Kepong | Lambertz et al. (2014) |  |
| *L. poilani* | DQ283378 | AMNH A163717 | Vietnam, Quang Nam Prov., Tre My Dist. | Frost et al. (2006) |  |
| *L. savan* **sp. n.** | MK688592 | FMNH 255388 | Laos, Khammouan Prov., Boualapha Dist. | This study |  |
| *L. savan* **sp. n.** | MK688593 | FMNH 255390 | Laos, Khammouan Prov., Boualapha Dist. | This study |  |
| *L. savan* **sp. n.** | MK688594 | FMNH 266149 | Thailand, Ubon Ratchathani Prov., Na Chaluai Dist. | This study |  |
| *L. savan* **sp. n.** | MK688595 | FMNH 266156 | Thailand, Ubon Ratchathani Prov., Na Chaluai Dist. | This study |  |
| *L. savan* **sp. n.** | MK688596 | FMNH 266158 | Thailand, Ubon Ratchathani Prov., Buntharik Dist. | This study |  |
| *L. savan* **sp. n.** | MK688597 | NCSM 76287 | Laos, Savannakhet Prov., Vilabouli Dist. | This study |  |
| *L. savan* **sp. n.** | MK688598 | NCSM 76288 | Laos, Savannakhet Prov., Vilabouli Dist. | This study |  |
| *L. savan* **sp. n.** | MK688599 | NCSM 76289 | Laos, Savannakhet Prov., Vilabouli Dist. | This study |  |
| *L. savan* **sp. n.** | MK688600 | NCSM 76291 | Laos, Savannakhet Prov., Vilabouli Dist. | This study |  |
| *L. savan* **sp. n.** | MK688601 | NCSM 76294 | Laos, Savannakhet Prov., Vilabouli Dist. | This study |  |
| *L. savan* **sp. n.** | MK688602 | NCSM 84943 | Laos, Savannakhet Prov., Vilabouli Dist. | This study |  |
| *L. savan* **sp. n.** | MK688603 | NUOL 00061 | Laos, Savannakhet Prov., Vilabouli Dist. | This study |  |
| *L. woodworthi* | JX911331 | TNHC 61942 | Philippines, Luzon Island, Quezon Prov., Atimonan Municip. | Oaks et al. (2013) |  |
